# Effects of *Siraitia grosvenorii* seed flour on the properties and quality of steamed bread

**DOI:** 10.3389/fnut.2023.1249639

**Published:** 2023-08-21

**Authors:** Wei Zhou, Siyu Guo, Sheng Zhang, Zhaodi Lu, Ziyi Sun, Yulin Ma, Jinxiu Shi, Hao Zhang

**Affiliations:** ^1^School of Food Science, Henan Institute of Science and Technology, Xinxiang, China; ^2^Key Laboratory of Aquatic Products Processing and Safety Control, Xinxiang, China; ^3^Engineering and Technology Research Center of Aquatic Products Processing and Quality Control, Xinxiang, China; ^4^Shaanxi Research Institute of Agricultural Products Processing Technology, Xi’an, China

**Keywords:** *Siraitia grosvenorii* seed, antioxidant, rheological properties, processing properties, steamed bread

## Abstract

*Siraitia grosvenorii* seeds are rich in abundant active compounds beneficial to human health. To clarify the digestion characteristics of *Siraitia grosvenorii* seed flour (SSF) and promote the use of SSF in the processing of functional staple foods, SSF was prepared, its composition and physicochemical properties were studied, and the processing characteristics of SSF-wheat flour were systematically investigated. The results showed that the torque curve and other parameters of the dough were significantly affected by the amount of SSF added. With the increase of SSF proportion, the water absorption showed an increasing trend, while the degree of protein weakening first weakened and then enhanced. At 20% SSF, the dough was more resistant to kneading. In response to an increase in SSF, the L* value decreased significantly, and the a* and b* values increased gradually, while the specific volume decreased gradually. Additionally, the hardness, adhesiveness, and chewiness of the bread enhanced gradually, while its elasticity, cohesiveness, and resilience decreased gradually. After the addition of 30% SSF, the inner tissue of steamed bread was more delicate. With an increase in SSF proportion, the predicted glycemic index (pGI) of steamed bread weakened markedly. Overall, these results showed that SSF, as a kind of food ingredient with hypoglycemic activity, can be used in the production of new functional steamed bread products. This study provides basic research data for the development of products containing *S. grosvenorii* seed.

## 1. Introduction

*Siraitia grosvenorii*, also known as longevity fruit, is a species of the cucurbitaceae and is mainly planted in Guangxi, Hunan, and Guangdong provinces of China. As a medicinal and food homolog (1), *S. grosvenorii* has been used in traditional Chinese medicine and beverages for hundreds of years in China, due to its biological functions, including lowering lipid level (2), lowering blood glucose level (3), improving immune functions (4), and anti-inflammations (5). The triterpenoid in this fruit have been used as low-calorie sweeteners in recent years. Moreover, it has been proved that it can be used as a safe food additive by FDA (Food and Drug Administration, USA) (6). In industry, triterpenoid compounds in fruits are generally extracted by water extraction, whereas the remaining seeds are usually treated as wastes. The *S. grosvenorii* seeds (SS) account for 40 to 50% of the whole fresh fruit weight (1, 7). There are still abundant active compounds in SS, with oil (48.5%), dietary fiber (38.3%), and proteins (8.7–13.4%) (8, 9); so, the utilization SS has high economic value.

At present, studies on SS mainly focused on the extraction of SS oil, the extraction and purification of polysaccharides, and their antioxidant activity (10–12). An HPLC analysis showed SS oil contained 12.5% squalene (9, 13). Squalene has the functions of body building and antifatigue and can treat liver diseases (14). As a high-quality plant protein resource, its emulsifying and foaming properties are better than those of soybean protein isolate (15). However, there hasn’t been a report on the use of SS in developing new staple food.

Steam bread is a staple food in East Asian countries, made from wheat flour, water, and yeast, followed by fermentation, shaping, and steaming (16). The traditional steamed bread products are primarily made with white wheat flour and have excellent sensory and cooking characteristics. But fine processing causes several problems, including excess macronutrients and inadequate micronutrients. As a result, the steamed bread has a lower nutritional value (17, 18). With the increasing pursuit of health, the addition of some functional active ingredients during the production of steamed bread is an effective way to provide health benefits (16). As SSF is applied during the production of steamed bread, a variety of functional ingredients can be used to meet consumer demand for healthy diets (19). At the same time, it provides a framework for developing functional staple foods. But as far as we know, there is no report on the application of SSF in steamed bread. In view of this, the chemical compositions and physicochemical properties of SSF were systematically evaluated in this study. Then, processing characteristics of the mixed flour (SSF-wheat flour) were studied, as well as the rules of regulating the appearance, texture, and starch digestion of SSF in steamed bread were clarified to further promote the use of SSF in the processing of functional staple foods.

## 2. Materials and methods

### 2.1. Materials and reagents

*Siraitia grosvenorii* seed was bought from Guilin Monk Fruit Corp. Wheat flour was bought from Wudeli Flour Group Co., Ltd. (Xinxiang, China). Yeast was purchased from Angel Yeast Co., Ltd. (Yichang, China). 2, 20-azinobis (3-ethylbenzothiazoline-6-sulfonic acid) diammonium salt (ABTS), rutin, and 1, 1-diphenyl-2-picrylhydrazyl (DPPH) were purchased from Sigma-Aldrich (St. Louis, MO, USA), while potassium sulfate was obtained from Shanghai Aladdin Biochemical Technology Co., Ltd. (Shanghai, China). Zinc sulfate, potassium ferrocyanide, absolute ethanol, petroleum ether, hydrochloric acid, sodium dihydrogen phosphate, and disodium hydrogen phosphate were acquired from Tianjin Damao Chemical Reagent Factory (Tianjin, China). All other chemicals were analytical grade.

### 2.2. Preparation of the *S. grosvenorii* seed

After removing the impurities, SS were ground by a laboratory mill (Bühler ALMB, Wuxi, China), which was then sifted through a 100-mesh sieve at 300 rpm for 200 s by a sieve shaking machine with 26GG and 7XX sieves (Bühler ALMC, Wuxi, China), and the flour was collected.

### 2.3. Analysis of routine composition analysis

The routine composition of SS was determined according to the Methods of Chinese National Agricultural Industry Standards. The moisture content analysis was carried out by the oven-preventive method (GB 5009.3-2016). The mineral content analysis was detected using the dry ash method (GB 5009.4-2016). The analysis of protein content was performed with a Kjeldahl apparatus (GB 5009.5-2016). The crude fiber content was evaluated by acid and alkali digestion (GB/T 5009.10-2003). The results of routine composition analysis were shown on a wet basis.

### 2.4. Measurement of polysaccharide content

The polysaccharide content was carried out according to the phenol-sulfuric acid method (20). Briefly, the glucose standard solution (0.1 mg/mL) was accurately taken into a test tube (0.0, 0.2, 0.4, 0.6, 0.8, 1.0, and 1.2 mL), and diluted to 2 mL with deionized water. An ice bath was used to chill the mixture after being added successively with 1 mL of 6% phenol and 5 mL sulfuric acid. The absorbance value (Y) was detected at 490 nm using a TU-1810D ultraviolet-visible spectrophotometer (Beijing Puxi General Instrument Co., Ltd.). The polysaccharide concentration (X, mg/mL) was calculated using the glucose standard curve (*Y* = 6.2775X + 0.0455, *r*^2^ = 0.9992), and the extraction rate was calculated using Equation 1.


(1) $$\mathrm{Polysaccharide}\mathrm{extraction}\mathrm{rate}\left(\%\right)=\frac{\text{CV}}{\text{M}}\times 100\%$$

Where, C, V, and M are the polysaccharide concentration of extract (mg/mL), the volume of extraction liquid (mL), and the seed flour quantity (mg), respectively.

### 2.5. Measurement of dough rheological properties

The rheological properties of the dough prepared from mixed flour containing different levels of SSF (0, 10, 20, 30, 40, and 50%) were evaluated by a Mixolab2 apparatus (Chopin, Paris, France), based on the “Chopin+” protocol, following its test conditions. The wet base was 14% as the reference; the surface speed was 80 rpm; the target torque was (1.1 ± 0.5) Nm; and the dough weight was 75 g. The standard test comprised of three processes as follows: (1) the dough was blended at 80 rpm for 8 min at 30°C; (2) the temperature of the dough was increased to 90°C at a rate of 4°C/min, and the temperature remained for 7 min; (3) the temperature of dough was lowered to 50°C at the rate of 4°C/min and remained for 5 min. The total test time was 45 min (21).

### 2.6. Preparation of steamed bread

According to GB/T35991-2018, steamed bread was prepared using mixed dough containing *S. grosvenorii* seed flour (SSF).

Dough kneading: one gram of dry yeast was mixed with an adequate amount of water at 38°C (According to the water absorption rate determined by the Mixolab2 apparatus. Then, 100 g of the mixed flour containing different levels of SSF (0, 10, 20, 30, 40, and 50%) was mixed with yeast solution in a K5SS blender (Whirlpool, Benton Harbor, MI, USA).

Fermentation: the fermentation of the mixed dough was cultured in a fermentation tank (Shengheng FJX-13, Guangzhou, China) at 37°C for 60 min, and the relative humidity (RH) was 85%.

Pressing and forming: the fermented dough was rolled with a dough press (Gap was adjusted to 5 mm) 10 times by a sheeter (Fuxing DMT-10Bdough, Longkou, China). Then the dough was divided into two blocks. Each block was kneaded to a bun shape and put into the steamer.

Wake up: the steamer and the treated dough were placed in a wake-up box for 30 min at 37°C and 85% RH.

Steaming: after steaming for 25 min and stewing for 5 min, the dough pieces were cooled for 1 h before analysis.

### 2.7. Analysis of color and specific volume

The instrumental color measurement of steamed bread was performed by using a chromatic aberration instrument (CR-400, Konica Minolta, Osaka, Japan). CIE-LAB color scale was used to evaluate the color of steamed bread, and L*, a*, and b* values (lightness L*, redness a*, and yellowness b*) was obtained. The volume measuring instrument was used to determine the steamed bread volume and the calculation of specific volume was performed using the following formula:


(2) $$\lambda =\frac{\text{V}}{\text{M}}$$

where, λ is the specific volume of steamed bread, mL/g; V is the volume of steamed bread, mL; M is the mass of steamed bread, g.

### 2.8. Measurement of texture characteristics of steamed bread

A standard probe (P/36R) was chosen to detect the texture profile (TPA, Konica Minolta, Osaka, Japan). The steamed bread was cut into 15 mm thick slices for TPA determination with the following parameters: pretest probe speed was 3 mm/s; test probe speed was 1 mm/s; posttest probe speed was 1 mm/s. The compression ratio was 30%, the compression time was 3 s, and the trigger force was 5 g.

### 2.9. Measurement of internal structure image

The middle part of a slice of steamed bread was taken and placed in the sample plate for image analysis of its internal tissues by a C-Cell food image analyzer (Caliber Control International Ltd., Warrington, UK).

### 2.10. Measurement of antioxidant activity and polyphenol content

Five grams of dried samples were used to extract the active ingredient with 180 mL of 80% ethanol by refluxing at 80°C for 7 h. The filtrate was collected after filtration, and ethanol was removed by rotary evaporation.

The DPPH radical scavenging ability was detected according to the method of Ngamsuk et al. (22). The extract solution of different concentrations (2 mL) were mixed with 2 mL of DPPH solution (0.2 mM), and the mixtures were kept in darkness at room temperature for 30 min. The absorbance of the samples was measured at 517 nm. The control group was a mixture of 2.0 mL DPPH solution and 2.0 mL ethanol. Determination of DPPH radical scavenging ability was done based on the following formula:


(3) $$\mathrm{DPPH}\mathrm{radical}\mathrm{scavenging}\mathrm{activity}\left(\%\right)=\frac{A\,\mathrm{control}-A\,\mathrm{sample}}{A\,\mathrm{control}}\times 100\%$$

A_*control*_: the absorbance of the control group; A_*sample*_: the absorbance of the sample group.

The ABTS radical scavenging capacity was estimated according to the method of Li et al. (23). ABTS free radical cationic solution was obtained by mixing 100 mL of ABTS solution (7 mM) and 1.75 mL of potassium persulfate solution (2.45 mM) and reacting in darkness at room temperature for 12 h. The mixture was diluted with 0.05 M phosphate buffer (pH 7.4) until the absorbance at 734 nm was 0.70 ± 0.02. And the diluted solution was used as the ABTS radical test solution. For ABTS analysis, 0.15 mL of the extract solution having different concentrations was added to 2.85 mL ABTS free radical test solution, which was kept at room temperature for 10 min; the absorbance of the sample (A_*sample*_) at 734 nm was detected. For the control group, 0.15 mL 80% methanol and 2.85 mL ABTS free radical test solution were used, and the absorbance was measured (A_*control*_). ABTS free radical scavenging ability was concluded according to the following formula:


(4) $$\mathrm{ABTS}\mathrm{radical}\mathrm{scavenging}\mathrm{activity}\left(\%\right)=\frac{A\,\mathrm{control}-A\,\mathrm{sample}}{A\,\mathrm{control}}\times 100\%$$

The analysis of total phenol content was assessed following the method of Hayes with slight modification (24). Accurately 25 mg of the gallic acid standard was dissolved in 250 mL water to make a standard gallic acid solution. The gallic acid standard solution (0.0, 0.1, 0.2, 0.3, 0.4, and 0.5 mL) was added to 10 mL of Folin–Ciocalteu chromogenic agent, then 2 mL of 10% Na2CO3 solution was added and the volume was made to 25 mL with deionized water. The absorbance value was detected at 765 nm after being kept at room temperature for 20 min. A linear regression equation was made according to the results above. The polyphenol was extracted by ultrasonic-assisted extraction. Briefly, 0.2 g of the extract was added to 40 mL of 60% acetonitrile, and the mixture was extracted for 20 min. The extraction was carried out twice, and the filtrate was collected. The absorbance of the sample was determined according to the above method, and the content of polyphenol was calculated according to the regression equation.

### 2.11. Analysis of *in vitro* digestion

Glycemic index analysis of steamed bread was performed with a NutriScan GI20 (Next Instruments Pty Ltd, Condell Park, NSW, Australia) (25). A simulation of food digestion through the mouth, gullet, stomach, and small intestine was performed. The details were as follows: in the oral stage, the freeze-dried steamed bread containing 50 mg starch was mixed with 2 mL of simulated saliva solution; the rotor was used to simulate peristaltic digestion for 5 min. After digestion, the simulated intestinal fluid (5 mL) was added and incubated for 1.5 h; then, the buffer was added to adjust the pH of the solution. Five milliliters of simulated intestinal juice was injected and hydrolyzed for 4 h. Glucose release was recorded by the built-in glucose analyzer at regular intervals; the pGI of each sample was carried out by the NutraScan GI 20 software.

### 2.12. Statistical analysis

Results are shown as mean ± standard deviation (*n* = 3). For significance analysis, the Tukey method was used with a 95% confidence interval. The Origin 8.5 software (OriginLab Corporation, Northampton, MA, USA) was used for statistical analysis and mapping.

## 3. Results and discussion

### 3.1. Chemical composition analysis

Table 1 shows the chemical composition of SSF. The content of protein and polysaccharide were 13.33 ± 0.14% and 6.87 ± 0.36%, respectively. In addition, the ash content and crude fiber content were 3.45 ± 0.12% and 34.31 ± 0.46%, respectively. SSF was rich in fiber. The fiber in food can promote the peristalsis of the gastrointestinal tract, help to digest and absorb food, improve the symptoms of constipation, replenish the nutrients needed for the body, maintain the normal operation of the body, and accelerate the metabolism of the body, enhance the body’s immunity. Therefore, SSF has a potential health improvement effect.

**TABLE 1:** Chemical composition of SSF (%).

Water	Mineral	Crude fiber	Crude proteins	Polysac-charide
6.74 ± 0.13	3.45 ± 0.12	34.31 ± 0.46	13.33 ± 0.14	6.87 ± 0.36

### 3.2. Rheological properties of dough

**FIGURE 1:**
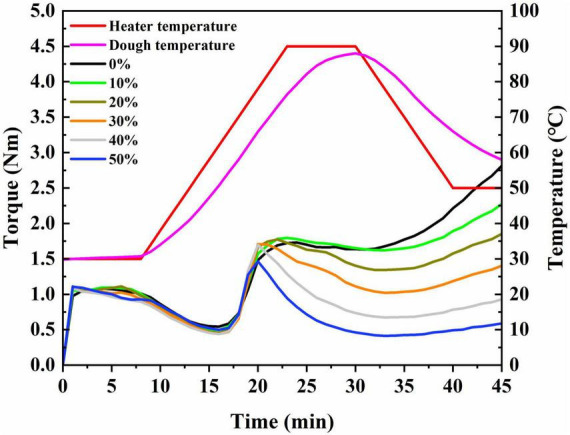
The Mixolab2 curves of the dough.

The effect of mechanical shear stress and temperature on the characteristics of the mixed flour was measured by Mixolab2. The thermo-mechanical characteristics of protein components included: water absorption, formation time, stabilization time, C1 (maximum torque value), C2 (the protein weakening degree), and α (the degree of protein weakening with temperature rise). The thermo-mechanical properties of starch components included C3 (peak viscosity), C4 (maintain viscosity), C5 (starch retrogradation viscosity), C3-C2 (gelatinization degree), C3-C4 (viscosity disintegration), C5-C4 (retrogradation value), β (gelatinization degree of starch), and γ (starch breakage degree). The thermo-mechanical characteristic curves of Mixolab2 of dough components with different mass fractions of fruit seed flour are shown in Figure 1.

From Figure 1 and Table 2, it can be observed that the dough torque curve and parameters vary with SSF amount. With different SSF addition ratios, dough absorbs water differently. With the increase in the amount of SSF, the water absorption rate also showed an increasing trend, which might be due to the water absorption of SSF being stronger than that of wheat flour. When the proportion of SSF was 20%, the dough formation time was the longest, which was 5.92 min. With the increased proportion of SSF, the dough stability time decreased notably. As the proportion of SSF increased from 0 to 20%, the dough became more resistant to kneading. The protein weakening degree lessened firstly and then improved with the proportion of SSF range from 0 to 50%, and the minimum protein weakening degree was observed at 20, 30, and 40% of SSF, with no significant difference, which demonstrated that the protein weakening degree of the mixed flour of SSF-wheat flour was the greatest. Additionally, a thinning reaction could easily be produced after dough formation, decreasing dough viscosity. Moreover, there was no significant change in C3, when the SSF was added at 0% and increased from 20 to 40% (*P* > 0.05). At 50% SSF, C3 was the lowest, indicating that the hardness and viscosity of the paste after gelatinization were the weakest. There was no change in C3-C2 and β when the seed flour supplemental ratio was 10 and 20% (*P* > 0.05), illustrating that the gelatinization characteristics were the strongest; gelatinization speed was the fastest; viscosity was the largest; and tissue adhesion was the best at this time. However, increased SSF caused C3-C4 to increase, indicating that the thermal stability of starch gelatinization of mixed flour decreased and the tissue adhesion was poor, while C5-C4 decreased with the increasing proportion of SSF. It was manifested that the higher the addition ratio, the less easy to regenerate.

**TABLE 2:** Parameters values of Mixolab2 curves of all samples.

Parameters	The proportion of SSF (%)					
	0	10	20	30	40	50
Water absorption (%)	63.3 ± 0.2^e^	63.6 ± 0.1^e^	65.3 ± 0.4^d^	68.8 ± 0.2^c^	73.4 ± 0.1^b^	78.6 ± 0.5^a^
Development time (min)	4.180 ± 0.036^b^	4.187 ± 0.625^b^	5.920 ± 0.105^a^	1.000 ± 0.044^c^	1.190 ± 0.121^c^	1.193 ± 0.078^c^
Thermal stability (min)	8.000 ± 0.173^a^	7.733 ± 0.058^a^	7.400 ± 0.100^b^	6.933 ± 0.321^c^	5.000 ± 0.100^d^	4.933 ± 0.058^d^
C1 (Nm)	1.100 ± 0.029^a^	1.104 ± 0.016^a^	1.119 ± 0.029^a^	1.099 ± 0.021^a^	1.091 ± 0.001^a^	1.121 ± 0.018^a^
C2 (Nm)	0.543 ± 0.010^a^	0.493 ± 0.011^b^	0.453 ± 0.015^c^	0.438 ± 0.006^c^	0.435 ± 0.016^c^	0.496 ± 0.017^b^
C3 (Nm)	1.738 ± 0.010^b^	1.811 ± 0.011^a^	1.782 ± 0.032^a^	1.736 ± 0.010^b^	1.705 ± 0.021^b^	1.487 ± 0.018^c^
C4 (Nm)	1.618 ± 0.002^a^	1.613 ± 0.016^a^	1.424 ± 0.153^b^	1.017 ± 0.012^c^	0.669 ± 0.008^d^	0.407 ± 0.007^e^
C5 (Nm)	2.850 ± 0.025^a^	2.284 ± 0.028^b^	1.860 ± 0.010^c^	1.404 ± 0.020^d^	0.931 ± 0.017^e^	0.591 ± 0.029^f^
C3-C2 (Nm)	1.195 ± 0.001^d^	1.318 ± 0.002^a^	1.329 ± 0.018^a^	1.297 ± 0.011^b^	1.270 ± 0.013^c^	0.992 ± 0.004^e^
C3-C4 (Nm)	0.120 ± 0.008^d^	0.198 ± 0.005^d^	0.358 ± 0.137^c^	0.719 ± 0.010^b^	1.036 ± 0.028^a^	1.081 ± 0.017^a^
C5-C4 (Nm)	1.232 ± 0.024^a^	0.671 ± 0.014^b^	0.436 ± 0.155^c^	0.387 ± 0.008^c^	0.263 ± 0.012^d^	0.184 ± 0.024^d^
α (Nm/min)	−0.076 ± 0.006^ab^	−0.069 ± 0.005^a^	−0.087 ± 0.009^b^	−0.065 ± 0.010^a^	−0.062 ± 0.004^a^	−0.067 ± 0.015^a^
β (Nm/min)	0.351 ± 0.084^b^	0.553 ± 0.020^a^	0.616 ± 0.058^a^	0.385 ± 0.012^b^	0.367 ± 0.093^b^	0.273 ± 0.057^b^
γ (Nm/min)	−0.018 ± 0.023^ab^	−0.006 ± 0.021^a^	−0.039 ± 0.014^bc^	−0.057 ± 0.020^c^	−0.052 ± 0.006^c^	−0.058 ± 0.013^c^

The different lowercase letters in the same row indicate remarkable differences (*P* < 0.05) (similarly hereinafter). C1, maximum torque value; C2, the protein weakening degree; C3, peak viscosity; C4, maintain viscosity; C5, starch retrogradation viscosity; C3-C2, gelatinization degree; C3-C4, viscosity disintegration; C5-C4, retrogradation value; α, the degree of protein weakening with temperature rise; β, gelatinization degree of starch; *γ*, starch breakage degree.

### 3.3. Color and specific volume of steamed bread

The color is an essential characteristic of the quality of steamed bread, which influences the appearance of a steamed bun. Moreover, it can affect consumer acceptance (26). Table 3 shows the results of instrumental color. With the increase of the additional amount of SSF, the L* value decreased significantly, while a* and b* values increased significantly (*P* < 0.05), indicating that the color of the steamed bun with SSF became dark and yellow by degrees, which might be due to the yellowness of SSF. Zhang et al. (21) found a similar phenomenon when studying the effect of Tartary buckwheat bran flour on the quality of steamed bread.

**TABLE 3:** The color of steamed bread.

SSF (%)	L* value	a* value	b* value
0	85.06 ± 0.72^a^	−5.92 ± 0.09^f^	13.57 ± 0.60^e^
10	72.21 ± 1.44^b^	−2.25 ± 0.14^e^	29.35 ± 0.26^d^
20	67.40 ± 0.31^c^	0.45 ± 0.07^d^	33.36 ± 0.46^c^
30	64.06 ± 0.65^d^	1.94 ± 0.20^c^	34.77 ± 0.62^b^
40	63.13 ± 0.35^d^	2.69 ± 0.12^b^	36.31 ± 0.24^a^
50	62.74 ± 0.62^d^	3.11 ± 0.21^a^	36.78 ± 0.11^a^

L*, a*, and b* are the proprietary parameters of the colorimeter. The different lowercase letters in the same column indicate significant differences (*P* < 0.05).

**FIGURE 2:**
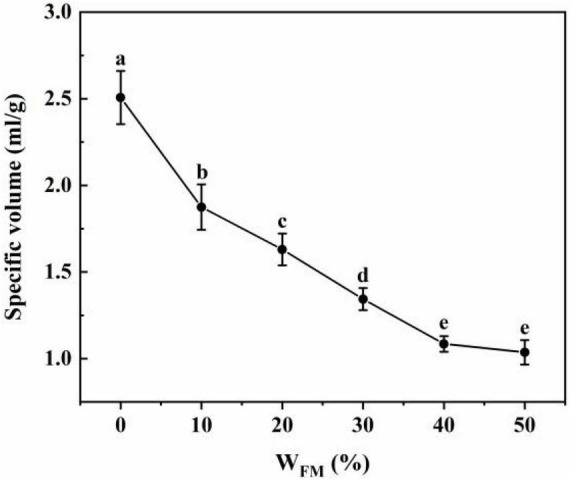
Effect of W_*FM*_ on the specific volume of steamed bread. The different lowercase letters in the figure indicate significant differences (*P* < 0.05).

Figure 2 illustrates how SSF content affects steamed bread volume specifically. There was a negative correlation between the percentage content of SSF (WFM) and the specific volume of steamed bread up to 40% of SSF, while there was no difference (*P* > 0.05) between 40% of SSF and 50% of SSF and the specific volume was 1 mL/g. This might contribute to the SSF-weakened wheat gluten and hinder the formation of a gluten network. Thus, the volume of steamed buns decreased as a result of decreased swelling force.

### 3.4. Texture characteristics of steamed bread

Chewiness and hardness are both crucial indicators to evaluate the character of a steamed bun (27). The different quantities of SSF addition on steamed bread quality and structure are illustrated in Table 4. The results showed that hardness, gumminess, and chewiness increase by degrees with the additive amount of SSF. This might be due to the addition of SSF damaging the gluten network, resulting in difficulty in repristinating. However, the elasticity, cohesiveness, and recovery of the steamed bread were all reduced.

**TABLE 4:** The texture characteristics of steamed bread.

SSF (%)	Hardness (g)	Springiness	Cohesiveness	Gumminess (g)	Chewiness	Resilience
0	2,457 ± 108^f^	1.000 ± 0.024^a^	0.862 ± 0.018^a^	2,111 ± 67^f^	2,106 ± 118^f^	0.590 ± 0.017^a^
10	3,555 ± 186^e^	0.972 ± 0.035^ab^	0.862 ± 0.010^a^	2,931 ± 250^e^	2,886 ± 184^e^	0.586 ± 0.011^a^
20	8,739 ± 209^d^	0.951 ± 0.005^bc^	0.805 ± 0.008^b^	6,929 ± 274^d^	6,590 ± 290^d^	0.515 ± 0.008^b^
30	13,617 ± 263^c^	0.916 ± 0.005^cd^	0.762 ± 0.008^c^	10,370 ± 174^c^	9,495 ± 145^c^	0.451 ± 0.010^c^
40	18,769 ± 291^b^	0.875 ± 0.002^de^	0.710 ± 0.014^d^	13,334 ± 280^b^	11,876 ± 277^b^	0.388 ± 0.013^d^
50	25,068 ± 174^a^	0.832 ± 0.042^e^	0.670 ± 0.014^e^	16,831 ± 294^a^	13,783 ± 251^a^	0.376 ± 0.022^d^

The different lowercase letters in the same column indicate significant differences (*P* < 0.05).

### 3.5. Internal structure of steamed bread

**FIGURE 3:**
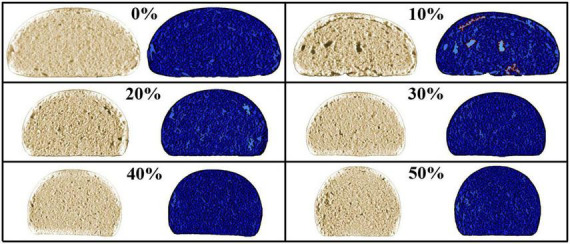
C-Cell cell images of steamed bread slices with the addition of SSF ranging from 0 to 50%.

C-Cell image analyzer was developed by the British Caliber Control International Company for fermented flour products’ quality control system based on computer technology. It works through the process when the image of the plane gets information about sample porosity and sliced characteristic parameters. The internal texture structure of fermented flour products can be comprehensively evaluated (28). Figure 3 displayed the C-Cell scan images of steamed bun with the addition of SSF, while Table 5 showed their C-Cell parameters. According to the results, we found that the sliced area and brightness of steamed bread decreased gradually following the addition of SSF. While the stomatal contrast, stoma number, porosity, and stomatal density increased and then decreased. The stomatal area, air wall thickness, stomatal diameter, stomatal volume, and coarse stomatal volume improved firstly and then lessened, indicating that the effect of the addition of SSF on the internal structure of steamed bread was first rough and then fine. The changing situations of brightness and whiteness coincided with the results of instrumental color, indicating that the interior sheen was consistent with the surface sheen of steamed bread and there was no remarkable difference when the additive amount exceeded 30% (*P* > 0.05). According to the changes of slice area, stomatal number, stomatal density, stomatal diameter, stomatal area, and stomatal volume, the size of stomata and the volume of rough stomata increased first and then decreased, indicating that the adding amount of SSF at 10 and 20% reduced the texture of steamed bun. The interior organization of the steamed bun became more delicate when the additional amount exceeded 20%. This might be due to that the air-holding capacity of the dough after the 20% addition was reduced in the process of waking up, which led to the steamed bread becoming firmer after steaming because of the presence of internal sand and granules, which increased the degree of delicacy. There was a negative correlation between stomatal contrast and pore wall thickness. The addition of SSF over 10% led to the thinning of the pore wall and the increase of stomatal contrast, which weakened the gloss of steamed bread. In addition, there was no significant difference in stomatal elongation at 0, 20, 30, and 50% additive amounts of SSF (*P* > 0.05), indicating that the stomatal flat length of steamed bread was the same at this time.

**TABLE 5:** The parameter values of Mixolab2 curves of mixed dough.

W_FM_ (%)	0	10	20	30	40	50
Slice area (mm^2^)	2,945 ± 104^a^	2,517 ± 35^b^	2,337 ± 12^c^	2,093 ± 80^d^	2,045 ± 9^de^	1,968 ± 22^e^
Brightness	124.302 ± 3.633^a^	79.277 ± 0.412^b^	69.058 ± 1.392^c^	59.328 ± 0.799^d^	57.300 ± 0.087^d^	56.812 ± 0.563^d^
Cell contrast	0.812 ± 0.001^a^	0.692 ± 0.002^d^	0.717 ± 0.008^c^	0.744 ± 0.021^b^	0.761 ± 0.007^b^	0.750 ± 0.020^b^
Number of cells	2,564 ± 70^a^	1,749 ± 45^d^	1,917 ± 104^c^	2,000 ± 64^c^	2,153 ± 83^b^	2,189 ± 106^b^
Area of cells (mm^2^)	45.771 ± 0.312^b^	46.667 ± 0.101^a^	46.359 ± 0.202^ab^	43.601 ± 0.285^c^	43.654 ± 0.308^c^	43.756 ± 1.007^c^
Wall thickness (mm)	0.417 ± 0.002^b^	0.452 ± 0.004^a^	0.425 ± 0.003^b^	0.403 ± 0.007^c^	0.390 ± 0.003^d^	0.386 ± 0.005^d^
Cell diameter (mm)	1.292 ± 0.054^c^	1.495 ± 0.048^a^	1.402 ± 0.029^b^	1.125 ± 0.034^d^	1.083 ± 0.029^d^	1.066 ± 0.059^d^
Cell volume (mm^3^)	3.265 ± 0.075^c^	4.912 ± 0.220^a^	4.374 ± 0.246^b^	3.195 ± 0.130^c^	3.054 ± 0.155^cd^	2.831 ± 0.231^d^
Coarse cell volume (mm^3^)	4.971 ± 0.058^c^	8.906 ± 0.065^a^	6.762 ± 0.241^b^	4.986 ± 0.364^c^	4.378 ± 0.144^d^	4.512 ± 0.507^cd^
Cell elongation (mm)	1.585 ± 0.004^ab^	1.546 ± 0.018^c^	1.575 ± 0.011^b^	1.603 ± 0.020^ab^	1.612 ± 0.025^a^	1.573 ± 0.003^bc^
Cell density (g/mm^3^)	0.871 ± 0.012^c^	0.695 ± 0.026^d^	0.820 ± 0.046^c^	0.957 ± 0.064^b^	1.053 ± 0.036^a^	1.112 ± 0.058^a^

The different lowercase letters in the same row indicate significant differences (*P* < 0.05).

### 3.6. Antioxidant activity and polyphenol content

**FIGURE 4:**
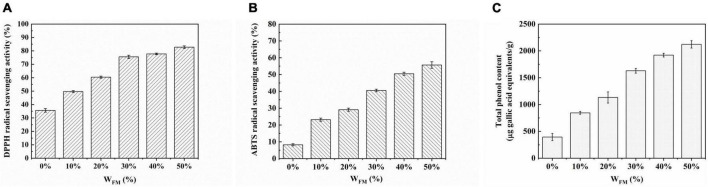
Effect of W_*FM*_ on the DPPH **(A)**, ABTS **(B)** radical scavenging activities, and total phenol content **(C)** in steamed bread.

In physiological and pathological processes, reactive oxygen species (ROS) are the main molecules generated by the body as a result of oxidative stress. Most ROS are produced in cells endogenously by their mitochondrial respiratory chain (29). Plenty of evidence proved that organisms always keep the balance of extremely low levels of ROS under normal circumstances, while its level in mitochondria during many diseased conditions, such as cardiovascular diseases, diabetes, tumors, and inflammatory tissues, was more senior than that in normal cells. The antioxidants extracted from the plant can effectively preserve the level of ROS in the body, which reduce oxidative stress and diseases (30). DPPH and ABTS are considerably steady free radicals synthesized artificially. Antioxidants would clean DPPH and ABTS, and the change in solution absorbance would indicate the antioxidants’ free radical scavenging activity. This study systematically evaluated the DPPH and ABTS free radical scavenging ability of steamed bread extract. As shown in Figure 4, the scavenging ability of DPPH and ABTS of steamed bun extract increased with the increase in SSF concentration and there were significant differences in the scavenging ability at the same concentration (*P* < 0.05). Polyphenols in the seed flour were responsible for steamed bread’s notable antioxidant activity.

### 3.7. Determination of pGI

**FIGURE 5:**
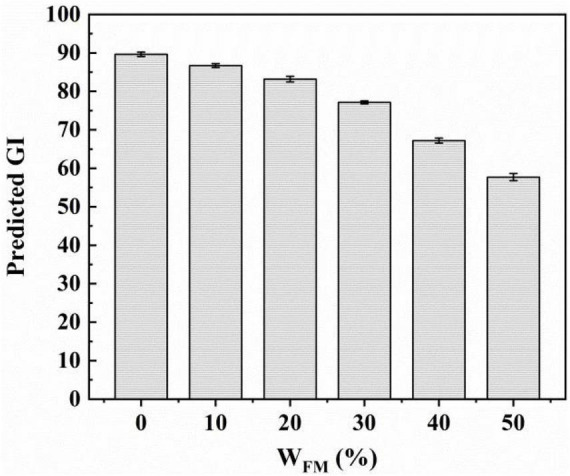
Effect of W_*FM*_ on the predicted GI value of steamed bread.

Food is evaluated by its glycemic index (GI) to determine how carbohydrates affect blood glucose levels. The blood glucose level rises rapidly when fast-absorbing foods (>70 GI) are consumed, while low-absorbing foods (55 GI) have the opposite effect (31). GI is connected to the starch structure, amylose level, and degree of crystallinity (32, 33). The foods with low GI are conducive to improving the blood glucose level of diabetics and also contribute to preventing cardiovascular diseases. Figure 5 showed the pGI value of steamed bread with different proportions of SSF. Increasing SSF proportions led to lower pGI of steamed bread (33). Its mechanism might be that the seed flour was rich in protein, which could have a certain wrapping effect on starch, and the wrapping effect might prevent the interaction of the amylase and the starch, so the hydrolysis of starch would be delayed.

## 4. Conclusion

In this study, the chemical composition of the SSF was analyzed and found to have abundant fiber and protein contents. SSF and wheat flour were combined to investigate the dough’s rheological properties. Then, the steamed bread based on seed flour and wheat flour was prepared. The peculiarities of the steamed bread were assessed, such as color difference and specific volume, texture characteristics, and internal structure. The results suggested that the addition of SSF had a significant impact on the properties of dough. With the increase of the proportion of SSF, the water absorption of the dough increased, and the strength of the protein first weakened and then increased. And the addition of SSF changed many characteristics of steamed bread. With the increase in the proportion of SSF, the color of bread darkened, the specific volume decreased, the hardness, adhesion, and chewiness increased, and the elasticity, cohesion, and resilience decreased. Besides, the addition of SSF could reduce the predicted glycemic index of bread. With the increase in the proportion of SSF, the blood sugar response of bread was weakened, which was helpful to control the blood sugar level. These results provided a new idea about the application of the SSF, as a food ingredient with hypoglycemic activity, to produce new functional staple food products.

## Data availability statement

The original contributions presented in this study are included in the article/supplementary material, further inquiries can be directed to the corresponding author.

## Author contributions

WZ: conceptualization, methodology, software, investigation, resources, writing, supervision, project administration, and funding acquisition. SG: methodology and validation. SZ: methodology, software, formal analysis, writing, and visualization. ZL and YM: validation and visualization. ZS: data curation. JS: formal analysis and data curation. HZ: conceptualization, formal analysis, investigation, resources, data curation, writing, supervision, project administration, and funding acquisition. All authors contributed to the article and approved the submitted version.
